# Ontological Transparency, (In)visibility, and Hidden Curricula: Critical Pedagogy Amidst Contentious Edtech

**DOI:** 10.1007/s42438-020-00198-1

**Published:** 2020-10-29

**Authors:** Michael Gallagher, Markus Breines, Myles Blaney

**Affiliations:** 1 Centre for Research in Digital Education, University of Edinburgh, Edinburgh, UK; 2 London School of Hygiene and Tropical Medicine, London, UK; 3 Information Services Group, University of Edinburgh, Edinburgh, UK

**Keywords:** Digital education, Critical pedagogy, Praxis, Automation, Higher education

## Abstract

The steady migration of higher education online has accelerated in the wake of Covid-19. The implications of this migration on critical praxis—the theory-in-practice of pedagogy—deserve further scrutiny. This paper explores how teacher and student-led educational technology research and development can help rethink online critical praxis. The paper is based on a recent research project at the University of Edinburgh that speculatively explored the potential for automation in teaching, which generated insights into current and future pedagogical practice among both teachers and students. From this project emerged a series of pedagogical positions that were centred around visions of the future of teaching in response to automation: the pedagogical potential of visibility and invisibility online, transparency, and interrogating the hidden curricula of both higher education and educational technology itself. Through the surfacing of these pedagogical positions, this paper explores how critical pedagogy can be built into the broader teacher function and begins to identify the institutional structures that could potentially impede or accelerate that process.

## Introduction

This paper presents pedagogical positions that emerged from a collaborative project exploring the role of automation in teaching in higher education. These positions suggested two findings. First, that emerging and contentious technologies such as automation can be used to stimulate a reconfiguration of pedagogical practice in ‘complex, dynamic, messy, political social and organizational contexts that are constantly changing and that will shape, and be shaped, by “digitalisation”’ (Jandrić et al. 2018: 3). This is an observation building on past research (notably Bayne 2015 and Bayne and Gallagher 2020) and one made even more complex, dynamic and messy by the onset of Covid-19. Secondly, these reconfigured pedagogical practices are possible expressions of a critical pedagogy, one which critically interrogates ‘the subsumption of life itself within digital technology and a “digitalist” rationality, and the naturalisation of this process’ (Lazarus 2019: 392) and how that can be employed alongside institutional values and expressed through critical praxis. The contentious technology acts as a provocation to imagine and articulate new pedagogical configurations, rather than any goal in and of itself.

Rather than new technologies being imposed on institutions, an imposition that carries with it a pedagogical logic of perpetually learning and unlearning educational technology (Ford 2019), the process of teachers, students, and staff co-imagining educational technology surfaces existing and emergent positions of pedagogical practice as well as the institutional values underpinning these practices. This paper emerges out of a project that attempted to enact such a process. The ‘Expanding the Teacher Function’ project (2019–2020) explored the potential role of automation in teaching. Through workshops and interviews with academics, staff and students at the University of Edinburgh, this community-driven approach to automation and educational technology research also surfaced institutional perspectives on pedagogy in the face of potential technological change. The research project reflects an ongoing effort to put the ideation and development of these educational technologies squarely in the hands of those tasked with using them. In this research project, several pedagogical positions emerged that have implications for critical pedagogy in the increasingly important online teaching space. Online teaching is transforming teacher-student interactions and it is therefore necessary to rethink critical pedagogy to consider how digital structures are reproducing biases and inequalities. The pedagogical applications being suggested in the following emerged from narratives of teaching and are expressions of the attributes of the teacher function by and for the University of Edinburgh, but also reveal emergent pedagogical positions around what teaching could conceivably be in some near future.

There has been increasing experimentation in higher education exploring how automation can ‘extend human capabilities and possibilities of teaching, learning, research’ (Popenici and Kerr 2017: 4). This is not an ahistorical experimentation but rather a continuation of educational technology increasingly performing portions of the work of the teacher and the student. Examples include assistive technologies, such as text to speech, predictive text, spell checkers, and more that have been broadly adopted in higher education. More recently, automation is increasingly emerging around student support (Myers et al. 2019), assessment, and curriculum review (Garg 2020; Chan and Zary 2019). There has been increasing experimentation with automation in the form of chatbots designed to perform some portion of the larger teacher function, generally in the form of asking questions, providing feedback, and performing additional administrative tasks associated with the teacher function (Sandu and Gide 2019). Automation that provides capacity for rapid group formation in courses, for pre-sessional instruction, and for surfacing student voice in curricular creation (Breines and Gallagher 2020) is being explored.

Ultimately though, automation in teaching surfaces ‘what it means to be multiply connected both in ecological terms and in machinic-artificial terms, and how that may change what it means to teach, what it means to be an educator, and what it means to be a student’ (Bayne and Jandrić 2017: 213). This change raises questions about if and how pedagogies need to respond, adapt, or potentially reject shifting engagements and uses of technology.

## Critical Pedagogy and the Digital

Pedagogy is positioned as the practices of teaching, and their manifestation in the student-teacher relationship (Smith et al. 2010: 3). Van Manen’s (2002) pathic pedagogical principles broaden this pedagogical scope by including the relational, emotional, moral, and personal dimension of the educational process. Building on this, critique of everyday life (a critical pedagogy) aims to ‘render ambiguities bearable, and to metamorphose what seems to be most unchangeable in mankind’ (Lefebvre 2002: 226). Affected by both dressage and education, learning mediates. Pedagogy is any practice, process, or experience that effects learning.

This theory-in-practice of pedagogy can be described as praxis: a conscious, skilled activity that can be understood by researching from the inside (Kemmis 2010). ‘Reflective practice’ (Schön 1987) and ‘scholarship of teaching’ are used to describe how teacher-researchers bring theory to bear on their practice and understand their practice in theoretical terms (Fanghanel et al. 2016; Trigwell et al. 2010). Freire (1970: 126) defines praxis in a more critical way as ‘reflection and action directed at the structures to be transformed’. This is especially relevant in periods of transformational change (Beetham and Sharpe 2019), which for ‘complex, dynamic, messy, political social and organizational contexts that are constantly changing’ (Jandrić et al. 2018: 3) is persistent.

Praxis as transformation is also highly relevant in surfacing the structural, political, and social inequalities encapsulated in access to and success in higher education. In this surfacing, the digital is part of a larger system of (in)equality. Ownership, access, and meaningful use of the digital become harbingers of access to and success in higher education. This positions universities largely as technocracies, systems of governance in which ‘technically trained experts rule by virtue of their specialised knowledge and position in dominant political and economic institutions’ (Fischer 1990: 17) underpinning knowledge hegemony in higher education as a result (Timmis and Muhuro 2019).

Praxis in this context looks to ‘metamorphose what seems to be most unchangeable’ (Lefebvre 2002: 226) in higher education and in larger society through critique. Farrow (2017) echoes this criticality in moves towards ‘emancipatory forms of knowledge, i.e. those that illuminate or deconstruct the economic and social circumstances within which a particular piece of knowledge is produced and understood’. Critical pedagogy can extend to the interrogation of these knowledge structures being increasingly created and mediated through the digital, echoing Freire’s (1970: 9) position that ‘liberating education consists in acts of cognition, not transferals of information’.

Beyond these critical positions of pedagogy is the role of the digital itself in challenging the social and spatial binaries that underpin these positions: teacher and student, human teacher and non-human teacher function, physical classroom, and digital space. The digital reconfigures expressions of the student-teacher relationship and the pathic principles that inform them. Indeed, equitable participation in an increasingly technocratic society requires pedagogies that reflect the knowledge that emerges from the ‘technoscientific’ spaces (MacDonald 2014) where education (and increasingly much of one’s social life) is increasingly performed. This requires pedagogical engagement with *civic participation* ‘in decision-making about the scientific and technological conditions in which we live’ (Haraway 1997: 50). Pedagogy engages with the practices of civic participation through the creation of space where deliberation, adaptation, and discourse around technologies can occur across traditionally segmented fields. Indeed, it is necessary for such spaces to exist as these ‘technoscientific’ issues increasingly impact culture and pedagogy itself (MacDonald 2014). Such a pedagogical position ‘assumes that students come into class with important social, cultural, economic knowledge and concerns and works to critique and build upon that situated knowledge’ (Angus et al. 2001: 197). The work of critical pedagogy in this respect becomes a means of drawing on this situated knowledge towards an interrogation of the technoscientific spaces that educational technology is increasingly structuring.

The work on critical pedagogy has sought to address a range of social inequalities and power structures, but the emerging digital structures of higher education are changing the ways in which biases and inequalities are being reproduced and amplified. As a result of these shifts towards the digital, there is a growing body of literature interrogating inequality and bias in educational technology and how it is impacting teaching (and in turn praxis). The ‘neutrality façade’ of technology as some malleable and ideologically neutral ‘tool’ of education (Mawasi et al. 2020) is being increasingly critiqued. The reconfiguration of the teacher function raises questions about the power relations that do the governing work (Ball 2016), especially as many new forms of technology seem to subject students to both teachers and automation. Increasing critical attention is being drawn to how technology and other knowledge production tools worsen representations (Noble 2018) and inequities for already marginalized people, reinforcing the relationships between racism, knowledge production, private enterprise, and power (Benjamin 2019). The pervasive data-driven ‘hidden architecture’ of higher education (Williamson 2018) is increasingly accelerating the ‘human-algorithmic decision-making’ practices and surveillance in higher education (Prinsloo 2017), subjecting education itself to potentially the same algorithmic misrepresentations as critiqued in Ball (2016), Benjamin (2019), and Noble (2018).

The role of critical pedagogy in this regard becomes again about surfacing these social inequalities and power structures through the development of a critical consciousness (Freire 1970), and to metamorphose, transform, or liberate them through action. The focus of the underlying research project discussed in this paper, automation, lent itself to this process of critical consciousness and pedagogical transformation. This transformation suggests the need for higher education to maintain ‘both a critical attitude toward the present and a political commitment to experiment with the coordinates of the future’ (Coté et al. 2007). How this attitude and commitment is expressed pedagogically is interpretable and therefore potentially diverse, again suggesting the need for diverse approaches to praxis.

## Studying the Future

The pedagogical positions being proposed in this paper emerged from the ‘Expanding the Teacher Function’ project (2019–2020) at the University of Edinburgh. In our research, the primary focus was the *teacher function*, the aggregation of human teaching, code, algorithms, and human-student agency (Bayne 2015), and how that might be expanded through the introduction of new forms of automation in teaching. Findings emerging from this project suggested that engagements with potentially contentious educational technology generated valuable insights about how teaching is currently understood at the University of Edinburgh and what it should or could be (Gallagher and Breines 2020).

This project deliberately attempted to move away from the instrumental and essentialist arguments that often frame educational technologies. These positions ‘either imbue technologies with inalienable qualities (essentialism) or posit technology as a neutral means for realizing goals defined by their users (instrumentalism)’ (Hamilton and Friesen 2013: 1). Rather, we adopted a sociomaterial position in this project to identify assemblages of humans and non-human actors (Latour 1996) and their enactments in practices (Hannon 2013: 169). More explicitly, we were exploring the possible assemblages of the teacher function (Bayne 2015) through possible teaching practice with automation framing these explorations. This position is one ‘that is not driven by desires to increase productivity or replace human teachers but by a pedagogical search for creating new knowledge and ways of being’ (Malott 2020: 366).

To initiate this pedagogical search and to surface these teaching practices, we adopted a community-driven approach where we focused on how new technologies can be introduced to inform teaching practice instead of pursuing an instrumental agenda of using technology to solve the challenges of higher education (Breines and Gallagher 2020). A series of design events (*n* = 14) ranging from a few up to 20 participants were run from July–December 2019 throughout the university.

These events were designed to provide space for the community to articulate responses to this emergent space of teaching with automation. In these events, we positioned this research project in the larger space of work at the university around educational technology, advanced the working definition of the ‘teacher function’ on which subsequent discussion and design activity would rest (presented in Bayne 2015 as an assemblage of human teaching, code, algorithms, and human-student agency), and presented a series of provocations of essentialist and instrumental approaches to educational technology. These provocations were drawn from past and current research by the authors, and include automated technologies to track attendance by students using GPS and/or facial recognition on campuses, wearable headsets that automatically record attention data from students, and automated tracking of student data to provide a sense of presence and community among a particular cohort. These provocations served as provotypes (provocative prototypes, a prevalent method in design research) to provide space to create meaning in an emergent context and then to connect that meaning to cultural values (River and Mactavish 2017) around teaching. These provotypes begin to capture how technologies ‘open up social spaces’ (Gromme 2016: 1008) by making available space to respond with alternatives.

This was followed by design activities where participants reimagined teaching practices, educational processes, or new automated insertions into the teacher function as basic prototypes. We also conducted a series of interviews (*n* = 15) from August 2019 to January 2020 with current students at the university and staff in managerial and teaching positions. The workshops and interviews surfaced pedagogical positions that might begin to define the role of teaching in increasingly ‘technoscientific’ spaces (MacDonald 2014), where civic participation (Haraway 1997: 50) is critical. For the purposes of this research, the university itself is positioned as a technoscientific space and critical praxis presented as a means of making possible that decision-making within it. Emerging from this process were a set of pedagogical positions. These included ontological transparency, (in)visibility, and a shift from low to high impact in online teaching. How and if these pedagogical positions are translated into contextually and disciplinary specific praxis will naturally vary across the many schools of the university.

There is much needed pedagogical work in developing critical awareness of the larger entanglements of the teacher function that amplify existing bias and marginalization, reinforce existing knowledge hegemonies, and adversely structure the sociotechnical spaces where teaching itself is increasingly performed. There is a need for a critical pedagogy that challenges these power structures, harnesses institutional agency for achieving emancipatory aims from them (Means et al. 2017:12), and ultimately transforms (Freire 1970) and metamorphoses them (Lefebvre 2002). The pedagogical positions discussed in the following sections do not explicitly achieve these aims. They merely provide a set of positions on which a critical pedagogy might be mapped.

## Transforming High-Volume Low-Impact Activity to High-Impact Low-Volume Teaching Practice

Mirroring its increasingly technocratic discourse, much of the focus in the implementation of educational technologies in higher education is about generating high-volume teaching. However, such an approach contrasts with the high-value and low-volume teaching that many teachers as well as students’ favour, routinely expressed as contact time (Bayne and Gallagher 2019). The reason for this is that high-volume teaching rarely translates into high value for either teachers or students. This view is consistent with learning and teaching strategy at the University of Edinburgh overall (2018) which emphasizes ‘accessible, high quality, and well-provisioned pastoral support’ alongside ‘assessment and feedback that delivers constructive and supportive dialogue’, and develops ‘the research and enquiry-led skills to support original research’ in its students.

Clearly, such objectives are like those of many universities, but by linking this to the automation of teaching in higher education, it emerges that this shift does not necessarily create an ideological shift. Rather, the underlying values remain the same but will be expressed in different way in increasingly sociotechnical educational spaces. In this research on automation, it emerged that many teachers were eager to find new ways to increase the volume of some aspects of teaching not perceived to be of high value and some saw automation potentially a vehicle for doing so.

Many expressed interests in how teaching could be shifted from high-volume and low-impact to low-volume and high-impact. Note in the following passage from a member of staff how this is expressed with a critical awareness of the dominant discourse around education (freeing up time, human capital) juxtaposed against the qualitative (human support, complex things that require a human conversation):

I think there are quite mechanical things that automation can help with. If we think beyond teaching and learning and into spaces where kind of form scanning, form filling type stuff, processing data, I think there’s a huge potential for automation there. And that’s kind of interesting because it has the potential to free up people to do other things. So there’s two ways of looking at automation, one is that it’s about replacing people and that’s a pure cost saving, and then the other is that it’s about being able to do something qualitatively better with the, I’ve gotta say, human capital we have. So if we are thinking about student support, for example, being able to automate aspects of data entry or that kind of thing, then free up people to actually deal with more complex things that require a human conversation. [Alice, Professional Staff]

Here, automation is seen as a tool that could potentially facilitate a reconfiguration in the pedagogical approaches where teachers’ specialism could be applied more specifically to support students to make a greater impact. This would allow for moving away from the repetitive and often time-consuming tasks that had less impact on students’ learning. Even though these expressions were not bound exclusively in this discussion of automation (nor are they particularly novel), our research participants routinely expressed the desire to reconfigure human teacher activity towards high-impact low-volume teaching practice. In this instance, the engagement with automation provided them a mechanism to reimagine teaching practice, or to enact teaching practice that has already been reimagined.

Administration has become an increasing part of being a university teacher. This trend has contributed to the expansion of the teacher function into administrative roles:

I think we do not have a good enough definition of what teaching is. I do not think it’s separate from admin. I think there’s some admin that is different from teaching but I think that a lot of what we do when we teach is repeat the same thing to different people over and over again. We tailor it slightly differently for different people but we are basically doing the same task or doing the same content. [Dan, Teacher]

Rather than pedagogical activity designed to metamorphose, transform, or liberate, much of what constitutes teaching reifies existing practice or knowledge structures. The slight adjustments, the same tasks or same content performed over and over again become the purview of automation, an observation that reinforces the initial statement of the lack of a sufficient definition for what teaching is. The repetitive tasks immediately following suggest what it *is not*, or *needn’t* be.

Teaching is sometimes thought of as a solitary activity, but it is already distributed in wider teams:

You could say at that point some elements of the teacher function are distributed among the team. But, you know, honestly, is that, again, is that too different to now? You have course teams, programme teams, you know, no programme of study is delivered by a single person, you have a number of people who bring subject matter, expertise, so why would not you have maybe somebody more integral who brings some technical expertise? We have librarians embedded in programme teams, providing information about, you know, particular collections or resources, so it might look a little bit like that as well. So that’s not very radical I realise. [Mary, Professional Staff]

This distribution of the teacher role suggests that the shift to automation in some aspects of the teacher function is not necessarily the great shift that it is sometimes portrayed to be, but rather a general expansion of actors, roles, technologies, and practices consistent with what we understand the teacher function to be, an assemblage of humans, code, and the like (Bayne 2015). Such an expansion potentially provides the opportunity for critical pedagogy to emerge, assuming the actors involved bear with them a critical consciousness (Freire 1970) and critical praxis across traditionally segmented fields (teachers, technical professionals, students, professional services) is made explicit.

Staff familiar with automation were generally enthusiastic about the opportunities for implementing it and did not see it as a threat to existing practices. Indeed, in the following, we see the surfacing of critical praxis: embodying ‘good’ learning and teaching and judgement in response to a diversifying set of perspectives.

Does it change anything about what good learning and teaching is? I mean, it should embody what we think good learning and teaching is, but does it change the nature of good learning and teaching? No, I do not think it does. And does it change anything about teachers’ judgement? No. I think it allows for a different range of interactions, and it maybe allows teachers to see a different view of their students. [Anita, Professional Staff].

Instead of seeing automation as a threat to their profession, it was becoming increasingly clear that automation of some aspects of the teacher function would allow teachers to use more time for what they are specialized in:

Edinburgh is renowned for having brilliant staff who are world leaders in their field. Why do I want that person sitting behind the desk, marking exam scripts? We’ve got brilliant academics who are world leaders in all of their fields right through the university, they should be in a class, talking to students, not marking. If we move to course work that’s automated, course work that has automated evaluation, and we happen to have chat bots that are there, what we are doing is we are creating an environment where we can say you come to Edinburgh, part of the deal of coming here is that the staff that we have, when they are doing teaching hours, those teaching hours you are in contact with them....The web is a great source of content, but the web is not necessarily a great source of educational experiences, and it’s those educational experiences we should seek to offer, it’s our staff that know how to create those experiences so that they are genuine and authentic, and it’s personal contact with those staff that is one of the most valuable things that we can cause our students to have, so we should be using evaluation automation and chat bots to take high volume, low value tasks off academics, and leave academics to do the low volume, high value tasks, the contact bits. [Chris, Teacher]

Although the capacity of high-impact low-volume teaching practice remains largely undefined, this quote illustrates a way forward where both teachers’ time and skills are used to support students in more critical ways. We see the emphasis again on care, contact, and the design of educational experience as the hallmarks of praxis. Indeed, the social was considered to be essential in implementing such technologies. Several staff raised care and support as essential aspects of teaching. Expressed as ‘a feeling like they [students] are supported one on one’, ‘an ethos of care’, a ‘duty of care’, and ‘support for the students’, each represents a shared imperative to reposition this high-value low-volume reconfiguration around a larger duty of care. The duty of care and its importance in the teacher-student relationship was consistently expressed by research participants across colleges and has emerged from previous research projects at the University of Edinburgh exploring near future teaching (Bayne and Gallagher 2019).

Perhaps paradoxically, many saw automation and its consistent presence as a mechanism that could help service a broader sense of care and a broader teacher function. This form of care could be expressed in the relationships between teachers and students, but also by setting up the structures in ways that take into account students’ needs.

## Humans Are Humans and Bots Are Bots and Why It Is Alright to Be Invisible

A central issue emerging from this project in the potential use of automation in teaching was ontological transparency, the assertion that the constitutive elements of the broader teacher function (human, automation, code, algorithm) maintain their categorical differences. In short, that it is clear when one is interacting with automation as opposed to a human teacher. Many referred to the need for clarity in terms of interactions because if students doubt who they are communicating with, it would erode the trust of students. This applies also in the use of automation in teaching as there is a risk of misleading the students in terms of their interactions with the teacher and institution. To avoid this, it is necessary to make it very clear that humans are humans and bots are bots, particularly those communicative robots, autonomous systems that serve the needs of human communication (Hepp 2020). It has also been argued that ontological transparency is needed around the tendencies to humanize automation. This has particularly been the case around the amplification of gender biases that occur in automation (discussed in Feine et al. 2020; McDonnell and Baxter 2019) as humanization often tends to reproduce gender roles.

Yet, this presents a context, prevalent in the data emerging from this study in a single academic institutional context, that runs largely counter to the more commercial actors, implementations, and imaginaries surrounding automation itself. These commercial implementations are examples of quasi-communication agents: ‘essentially a matter of attributing communication to a machine and not communication in the sense of human symbolic exchange as theorized in symbolic interactionism’ (Hepp 2020: 4) with, in some cases, additional automated emotive functionalities (animated expressions emerging from the screen, for instance, that indicate the emotional state of the automation itself). The study discussed in this paper is a contrast here: there was clear indication that this automation should not be replicating emotive or perceived ‘human’ traits and should clearly identify itself as automation. This presents contrast to earlier experimentation even within higher education. For example, the Jill Watson experiment (2014) at Georgia Tech University which inserted automation into the teacher function as a teaching assistance role, noting that humans will tend to interact with automation as if it were human, even if they know otherwise, and further noting the potential threat of deception here (Eicher et al. 2018 December). Ontological transparency in automation sits in contrast to this, making no attempt to obfuscate the precise attribute of the teacher function: humans are humans and bots are bots.

Yet, the boundaries need to be clear for the students to maintain positive relations and not feel like they are treated as people who can be managed by robots:

I think all of our students want a relationship with the people who teach them. They all want to feel part of a community and want to feel some level of, I would imagine, reassurance that they are kind of doing okay. So I think if it felt like it was about replacing the teacher in some sort of timesaving, cost-efficiency way, and it felt like a lesser experience, then I think it would not sit well with students. If it’s very obviously adding something, so you can get something from automation when I’m not around. So when I am around, I will help, when I’m not around, there is a lesser version of help available, but there’s something available. And also, again, if it’s very clear to the students that whatever automation agent they may be using has been informed by, or programmed by, or designed by an academic, I think that’s really important too, because again, you are giving that message that, you know, it’s me, but it’s not me. [Alice, Professional Staff]

The research suggested that the integrity of the pedagogical position demands an ontological transparency that established clear distinctions between interactions with humans and with robots. This meant that it is not only necessary to distinguish between what is human and what is automation, but also to reveal the relationship between the automated agents and the humans:

I think it’s really important to say yes, this is automated, yes, this is computer generated, but it’s computer generated using rules I wrote, my judgement about you, and the feedback I would give you if I was sitting across the table from you. You’re not saying I’m offloading to some machine that does not care, you are saying I have designed some help for you, or have designed something for you to help you learn better. So there’s something about how we communicate it, and there’s something about making sure that it is about embodying some expertise and judgement from the teacher, and that that’s clear to student. [Mary, Professional Staff]

This points to the idea of ontological transparency. While automated agents were considered helpful, their value was dependent on being part of a system where their role and level of automation would be clear to the students, a point that was echoed in the student data:

I think it becomes an ethical issue if you have that, that bot posing as the instructor...the student may feel duped but also the instructor will be…someone else’ll be speaking on their half without it necessarily being the instructor saying something which could make them lose their credibility... But I think if we are, we are transparent that it is an avatar, it’s a digital being, I think, I think that’s fine...I do not even think it would cross their minds for the most part. If they realised that it is a…an automated system, either they’ll just disregard it if they do not like it. [Gossy, Student]

Transparency extended to other aspects of the teacher function. Through the increasing datafication of higher education, concerns are emerging among university staff and students about the protection of data. As a result, teachers and students raised other forms of transparency as deserving critical attention. This included transparency of the underlying data regimes and transparency of what these data regimes expose students to as well as the temporal dimensions of the data (when and how is it expunged from the University infrastructure and its collective memory):

There’s a dialogue to be had with the learner because they may feel that their data is going away to be processed somewhere, and then to be analysed in depth. So there’s a discussion to be had there, and that perception of the learner about whether they are being spied on, or whether the computer system is allowing them to not be spied on is going to come down to the dialogues that’s had with the learner, and it’s a whole lot less to do with the technology...Should you make sure that a human is looking at the interactions a human is having so that you can detect a student that’s not participating and then go and find out if they are okay, or check what’s going on before they waste a bunch of fees or have a mental health issue that gets out of control, or is it so important that the student is able to explore freely without anybody seeing the outcomes, that that data is kept private? So, there are questions there that cannot be answered by the technology alone. [Dan, Teacher]

These issues illustrate that automation cannot operate fully on its own but is highly dependent on human control. While this raises new issues in terms of privacy, these are issues that need to set up in a way that is transparent to make it clear to the students how they are seen and how the data are used. Such challenges can then be overcome with transparency and clarity about the human role in these systems.

Transparency can also be understood in relation to concerns over visibility, which also emerged in a range of ways in the research. Largely, this was an expression of data visibility: control, security, and exposure to third party commercial services. Yet, there were several instances of the direct pedagogical application of invisibility being expressed in the data, such as the creation of places to ‘explore without being observed’. This position of (in)visibility largely aligns with research emerging on the social value of anonymity online (Bayne et al. 2019) suggesting the need for approaches to teaching that acknowledge the increasing datafication of education and provide an alternative narrative to that increasingly pervasive data-driven ‘hidden architecture’ of higher education (Williamson 2018).

There was a wariness and/or fatigue towards this datafication, which invisibility might conceivably mitigate through the development of spaces of anonymous, unassessed exploration. This was explained by an academic who was keen to use automation in his own teaching:

…experiences that would happen outside of human contact would have an opportunity to do things that could be very positive for students, like letting them explore without being observed to be stupid. You know how people are worried about looking silly. If they can explore and the computing system is there to give them guidance and feedback to some extent, the automated systems could give fairly broad and course feedback at the moment, and as we work on these systems, the feedback will improve over a number of years. [Francis, Teacher]

Indeed, many of the specific applications of automation suggested by participants discussed in Breines and Gallagher (2020) are explicitly designed to activate that invisibility. The opportunities for students to explore ideas freely and not be concerned about being seen by other students, teachers, or a pervasive data architecture have a potentially significant pedagogical impact. Its expression in the context of this study (as invisibility) suggests a critical consciousness; the alternatives suggested (realizing that invisibility through design or practice) suggest a critical praxis.

However, such invisibility takes considerable institutional agency to refrain from datafication. Existing educational provision and global performance indicators are often mobilized towards statistical normalization, but the transparency we are proposing here is more directed at reconfiguring the teacher function towards a diverse array of teaching practices that put teacher-student or student-driven interactions at the centre, even if that student is invisible, even if that activity ‘does not count’. On some level, such movements between visibility and invisibility are nurturing the value and relevance of non-quantifiable ways of knowing (Markham 2019) towards critical praxis.

At the same time, many teachers and students emphasized the importance of the humanness of these interactions. There was a persistent focus on meaningful contact between teachers and students to surface the visibility of educational exchanges, or to surface the humanness of these exchanges. There was mention of the importance of removing invisibility at times because many considered that anonymity would lead to a lack of accountability, however defined. Visibility becomes a contested practice of critical pedagogy in this way. This suggests potentially a diversification of pedagogical practices that stimulates movement between visibility and invisibility. For invisibility, this involves capturing the social value of anonymity online (Bayne et al. 2019) through anonymous discussion and collaboration as well as the creation of space that allows for exploration without being observed. Additionally, embracing invisibility becomes an issue of critical praxis as we have the opportunity to advance alternative narratives to the increasing surveillance of teacher and student activity ‘by recognising the value of the sensibilities of anonymity, ephemerality and unreachability’ (Bayne et al. 2019: 65).

## Hidden Curriculum

It is important that despite the presence of transparency and invisibility, there will always be aspects of teaching that are hidden. The distinction between hidden and invisible is important here. The former suggests a deliberate or accidental concealment of tacit learning; the latter is, or can be, liberatory in freeing one from the surveilled and assessed spaces of teaching and learning with digital technologies. Surfacing the hidden-ness of education is very much the purview of critical pedagogy and very much provoked by the contentiousness that automation in teaching suggests.

A hidden curriculum has often been a ‘side effect’ of education. The transmission of norms, values, and beliefs conveyed in the classroom and the social environment has made education a space where ‘[lessons] which are learned but not openly intended’ (Giroux and Penna 1983). Margolis (2001: 22) equates this hidden curriculum with the production or reproduction of ‘social relations like race and gender hierarchy, social class reproduction, the inculcation of ideological belief structures’.

Edwards (2015: 268) referred to hidden curriculum as an implicit process relating to other structures, pointing out that the term is primarily used to criticize ‘educational institutions for reproducing implicitly the unequal opportunities, inequalities and exercises of power in the social order’, suggesting to many that higher education is ‘not for them’. Illich (1973: 51) argued that the hidden curriculum of education alienates the critical pedagogue forcing ‘us down pathways functional to the perpetuation of the existing order rather than allowing the pursuit of avenues which call out to us as particular subjects’. In short, the hidden curriculum becomes the focus of the transformation, liberation, or metamorphosis that critical pedagogy is employed to challenge.

The digital in this instance complicates this. It effectively amplifies the hidden curriculum of higher education and imposes a hidden curriculum itself: ‘the responsive software architectures of digital media are our new hidden curricula, reschooling adults and children alike in new modalities of knowing, perceiving, and acting’ (Adams 2017: 238). While there is broad recognition of the existence of (both digital and educational) hidden curricula and their significant social impact in terms of reproducing privilege and inequality, there has been less attention to the ways in which teachers can critically challenge these structures and what role the digital (which in this project is represented as automation) has in that process. There are also opportunities to arrange digital or social structures in ways that shape hidden curricula in ways that are potentially transformative. As such, pedagogy is not merely about teachers’ practice, but a critical consciousness of the impact of design and code on enforcing the existing order.

It is clear to many teachers that they are not only there to ensure that students learn domain-specific knowledge, but transformative practice:

I think that a lot of the people who would be giving lectures here would probably imagine that they have two roles, one is to get the students to have certain technical skills, so that’s specific to the discipline, and the other is to get the students to be thinking mathematically, and whatever type of creativity that eventually leads to. And I think that we are quite good at supporting the first part of that. I’m getting those technical skills, and so somebody in the end will be able to do such and such a thing, as they have demonstrated in an exam, and we are much vaguer about the second part, and I’m not sure that we necessarily have, particularly on the more theoretical parts of the discipline, a very good grasp of really how to do that very well. (Dan, Teacher)

The different aspects of teaching are manifested in teaching practices as well as the spaces and structures that facilitate teaching. Some of the skills that students learn emerge from these spaces and structures rather than from the teachers themselves, suggesting critical pedagogy in this instance is not only direct contact, care, and humanness, but a careful orchestration of structure itself. This recognition can be used to enable teachers to take greater control of the structures and thereby begin to interrogate the hidden curricula.

Through the recognition of where and how hidden curricula exist and how the inherent biases impact students, new opportunities for building critical pedagogy into these structures emerge. In relation to our consideration of the possibilities for automation in teaching, it became clear that the teacher function relies heavily on a range of forms of support that are partly hidden:

I’m looking at it in terms of what it is that the library staff do, that the librarians do because they are doing support. I would not necessarily call it teaching but they are doing support about, around information literacy, around finding information, evaluating it, comparing it, synthesizing it. Appropriately citing their sources and producing original pieces of work and the ethics of, of that. And there is scope for people to have more awareness and understanding of those processes. At the minute I think a lot of it is tick box. So that’s something that we are working on making the kind of teacher function of the librarian more meaningful and impactful rather than it just being a signposting role where we go, ‘oh by the way, here’s a demonstration of this database. It’s more like moving towards here is a discussion of the various merits and demerits and limitations of this particular database or whose voices might not be heard in this particular conversation due to socio-political factors. (Regina, Professional Staff)

The above passage suggests two points. First, there is a critical awareness to move away from the performative aspects of this information literacy instruction engendered by normalized practice (‘a lot of it is tick box’, a ‘signposting role’) and towards a diversification (‘surfacing voices that might not be heard’), both challenges to the existing hidden curriculum. Furthermore, this passage points to how the teacher function is closely enmeshed in the broader university structure and that critical pedagogy alone is not sufficient. Rather, it is necessary to build critical pedagogy into the structures that broadly constitute the teacher function. The new pedagogical positions relating to the shift from high-volume low-impact activity to high-impact low-volume teaching practice and the importance of ontological transparency in such teaching provide an opportunity to both critically surface and reimagine the hidden curriculum.

However, it is rather necessary to let the teachers and students guide this process as the automation function acts as pedagogical embodiment of the teacher themselves:

Because the conversation would need to be designed by the teacher, and by somebody with subject matter expertise, I think it’s another good example of where automation is extending the teacher function or enhancing the teacher function. It’s back to this idea of, you know, it’s not replacing a whole person, it’s maybe just doing part of the job of that person. And it’s being programmed by that person, so to some extent it’s an embodiment of what that teacher wishes to teach. I think that’s actually where some of the tension in the ed. tech space can be, that in a number of the scenarios I’ve seen, that it’s not teachers who are informing the design and operation of these systems. So in the surgical example, yes, a technical person would programme the robot, but as I say, they would programme it to a specification from an expert, a surgical expert. We do not seem to do that in ed. tech, we do not seem to say teachers are the experts, let us get some information from them and then let us, you know, programme according to, it seems to be much more kind of ed. tech, IT people coming in from the outside and going, ‘Education’s broken, let’s…’ (Gail, Teacher)

It is only through approaches as outlined in this passage that it becomes possible to develop the structures of teaching with a critical pedagogy. By allowing teachers to build critical thinking into the technological structures of teaching, by building critical practice into the design of automation itself, critical praxis is possible. This suggests the need for teachers and students to be critically ‘informing the design and operation of these systems’ explicitly, or to have essentially teacher and student-led educational technology research and development (a process discussed in Breines and Gallagher 2020). Creating a multitude of educational sites within the university to deliberate, adapt, and design through participatory models becomes a component of critical praxis, one that potentially begins to interrogate and potentially mitigate the more entrenched aspects of hidden curricula.

## Challenges and (Re)imagining

The bulk of this paper has been spent exploring the ways in which institutional responses to educational technology *suggest* the emergence of critical praxis. This paper does not, however, suggest the probability of this emergence or even the impact of its employ. Critical pedagogy itself is challenging. Transforming, liberating, or even metamorphosizing the hidden curricula at even a single institutional setting is even more challenging. The teacher function being increasingly bound in a broadening range of actors and technologies presents a particularly complex assemblage, as any adaptation of one impacts the collective function.

Yet, pedagogical positions emerged in this research that are potentially generative in realizing some measure of transformation: high-impact low-volume practice, ontological transparency, and (in)visibility. These positions are not critical pedagogy themselves, but rather nascent expressions of critical pedagogy in the sociotechnical spaces in which education is increasingly being performed. These pedagogical positions are means to critically adapt to the structural and societal shifts in which higher education is increasingly becoming enmeshed.

Institutionally and in this project, automation provoked a discussion that surfaced values, made explicit the knowledge mobilities of the university (Gallagher and Breines 2020), articulated possible applications of automation in teaching (Breines and Gallagher 2020), and in this case, suggested a set of pedagogical positions that inform critical praxis. Such an emphasis on critical practice is deliberate as ‘change to ... society must also reside in social practices rather than merely in the structures or values of agents’ (Heikkurinen 2018: 1657). Yet, this change is the purview of both the teacher and the institution. The reimagining of pedagogical practice is not found ‘in its abstraction as a learning process’ but rather as a collective social process (Dyke and Meyerhoff 2018), one that engages with the ‘embodied arrangement or composition of desires and creativity as territorialized through and by relations between people in motion’ (Shukaitis 2009: 14).

In this instance, automation served as a provocation to surface and engage with some of this ‘territory’ towards reimagined teaching practice. This is a taxing process precisely because it is perpetually reflective ‘on our experiences within and across our movements, paying particular attention to tensions between pedagogies that reproduce the education imaginary and, alternatively, pedagogies of refusal and reimagination’ (Dyke and Meyerhoff 2018: 175). Pedagogical reproduction, refusal, and reimagination were all presented in this project and even extended to students. In its simplest form as suggested by one teacher is the agency to accept, reject, or subvert the automation itself. In more sophisticated iterations, this might render student programmable automation, potentially mitigating against the amplification of the hidden curriculum and teacher and student marginalization. Critical praxis emerges from agency in this respect, having the capacity to choose.

There is a need to resist efforts to normalize the diversity of praxis that might emerge from a such a diverse range of actors, interactions, and (critical) pedagogical practices, a resistance against institutional ‘systemic functions for disciplining, policing, and protecting of the status quo’ (Dyke and Meyerhoff 2018: 177). This is not to inevitably pit the critical praxis of teachers against the institutional structures of governance and the technological structures that beget particular normalized outcomes, but rather to note their impact on sustaining a context whereby a diversity of approaches is encouraged.

To conclude, it is important to note that this paper does not suggest some probability that critical praxis will emerge in response to educational technologies that potentially reconfigure the teacher function. Praxis informed and shaped largely by managerial concerns is just as likely to emerge, particularly if there is little critique of the instrumental discourses in which these technologies are generally presented. We merely suggest that within each educational technology , there is opportunity to surface critical praxis.

There is a further need to reflect on whether the observations presented in this paper are contingent on the conditions of this specific university. Each university undertaking such a process of engaging with automation in this way would conceivably surface different narratives of teaching and different responses to how the teacher function could conceivably be redefined in response. Whether a set of pedagogical positions would emerge from the provocation that automation poses is likely to depend on institutional constraints and the time and space for those invested in the teacher function to develop praxis. Ultimately though, critical pedagogy cannot be assumed to exist in these educational entanglements that technologies such as automation are increasingly structuring. Critical pedagogy is deliberate, it adapts, and it may require provocation to surface.
